# Simulation analysis of pressure relief effect of upward mining in the lower protective layer (group) of deep extra-thick coal seam

**DOI:** 10.1038/s41598-025-02201-w

**Published:** 2025-05-19

**Authors:** Guangpeng Qin, Zhenyu Zhang, Jing Cao, Jinpeng Ma, Xueyuan Zhang

**Affiliations:** 1 College of Resource, Shandong University of Science and Technology, Tai’an, 271000 China; 2 National Engineering Research Center of Coal Mine Filling Mining, Shandong University of Science and Technology, Tai’an, 271000 China; 3 College of Finance and Economics, Shandong University of Science and Technology, Tai’an, 271000 China

**Keywords:** Deep mining, Protective seams (groups), Upward mining, Unloading, Extra-thick coal seams, Engineering, Software

## Abstract

In this paper, an examination of the upward mining of multi-protected layers through simulation is conducted in a mine in Dongsheng Coal Field, China, as an example. FLAC3D numerical simulation software was used to calculate the mining scheme of the lower protected layer (group) and analyze the stress changes and pressure unloading characteristics of the protected layer during the mining process. Similar simulation experiments were conducted to provide additional explanations on the characteristics of fissure development in the overburden rock layer and the expansion and deformation characteristics of the protected layer during the mining process. The test results show that the stress distribution curve of the protected layer can be divided into four parts: the original rock stress area, the stress concentration area, the stress reduction area, and the stress recovery area. The bending deformation of the coal seam is evident after the completion of mining the lower protected seam group, and the expansion deformation area undergoes three processes: increasing, decreasing, and stabilizing. Overall, the unloading rate and length of double-protected seam mining are much larger than those of single-protected seam mining, providing an engineering basis for the mining of deep extra-thick coal seam protected seam (group).

Coal has always been the first major energy in China ‘s primary energy consumption structure. With the increasing depth of coal mining in China, the number of mines with prominent risks in coal mines has also increased^1–4^. At present, the commonly used measures to prevent and control coal and gas outburst in China mainly include mining protective layer, pre-drainage of coal seam gas, hydraulic fracturing and permeability improvement^5–10^. Because the protective layer mining technology has a significant effect on the effective permeability and pressure relief of multiple coal seams and the realization of large-area outburst elimination, China has taken the mining protective layer as the main measure to prevent and control mine dynamic accidents in the ' Coal Mine Safety Regulations ' and the ' Coal Mine Rock Burst Prevention Supervision Guidance Manual ‘^11–14^. The research on protective layer mining technology in China mainly focuses on single protective layer mining, and the research on protective layer group under complex coal seam group conditions is not deep enough. In order to effectively prevent coal and gas outburst and improve the recovery and utilization rate of resources under the condition of improving the pressure relief rate of protected layer, multi-protective layer mining has become an important research content of coal mine safety mining.

In the past ten years, many experts and scholars at home and abroad have made important contributions to the research of protective layer mining technology. Academician Qian et al.^15–17^ put forward the theory of key strata of overlying coal rock deformation and the theory of masonry beam through many theoretical studies and field practices. Based on the theory of masonry beam, he studied the distribution law of cracks in mining coal rock mass, and put forward the theory of ' O ‘ring and the theory of key strata. As an effective simulation method in engineering, the construction of numerical model and similar physical model is also widely used in the study of protective layer mining. Shi et al.^18^ used RFRA application system to simulate and analyze the variation law of the thickness of the protected layer, the horizontal deformation characteristics and the influence of the relative layer spacing on the protected layer during the mining process of the single-layer protective layer. Zhang et al.^19^ summarized the stress evolution and deformation law of overlying coal seam in the upper part of the stope after the completion of protective layer mining through flac3d numerical simulation. Zhang et al.^20^ studied the delineation and influence of the protection range of the lower protective layer mining by using the finite element analysis software ANSYS. Ma et al.^21^ studied the protection effect of composite protective layer mining in gently inclined thick coal seam by combining numerical simulation with similar simulation experiment.

In China, the mining of protective layer is mainly divided into single protective layer mining and multi-protective layer mining. Among them, the single protective layer mining technology has been playing an important role in effectively increasing permeability and pressure relief of multi-coal seams and realizing large-area outburst elimination in China. Duan’s^22^ 9-parameter three-dimensional top-coal caving surface model established by drilling method can accurately estimate the top-coal caving angle on site, which provides a reference for the effective recovery of coal resources in the process of single protective layer mining. Based on the protective layer mining technology, Gao et al.^23^ proposed an effective method to solve the high mining stress problem of large space roof structure in mechanized mining of thick coal seam. Through the combination of numerical model and similar physics, Gao et al.^24^ clarified the movement law of overlying strata and the change characteristics of mine pressure after single protective layer mining, and clarified the evolution law of stope stress and rock displacement during the mining process of liberated strata. In the study of multi-layer protective layer mining technology, Song et al.^25–28^ and other scholars discussed the multi-layer protective layer mining method on the basis of analyzing the mechanism and feasibility of upward mining in close-distance coal seams. Fang et al.^29^ conducted a similar material simulation test on the repeated mining of long-distance double upper protective layer based on the background of Pingmei No.8 Mine, and the research results enriched the theory of long-distance multi-upper protective layer mining. Li et al.^30^ used the downward mining method to mine the double lower protective layer, and verified the effect of the double lower protective layer mining through the permeability coefficient.

In summary, domestic and foreign experts and scholars have studied the single protective layer more thoroughly, but there are few studies on the mining protective layer group under the condition of complex coal seam group. Taking a mine in Dongsheng Coalfield as an example, this paper mainly studies the evolution law of stress change, pressure relief characteristics, expansion deformation and fracture characteristics of the protected layer (group) in the process of multi-protective layer mining by using the upward mining method under the condition of coal seam group occurrence. By using the fast Lagrangian analysis of continua (FLAC3D)^31–35^numerical simulation, the stress variation law and pressure relief characteristics of the protected layer in the process of mining the protective layer are analyzed. Then, the method of constructing similar physical model^36–38^ is used to simulate and analyze the process of mining the protective layer, and the expansion deformation and fracture development law of the protected layer are studied. According to the calculation results of the numerical model and the experimental results of the similar physical model, the protection effect of the protected layer in the mining process of the protective layer group is studied in detail, which provides the engineering basis for the mining of the protective layer (group) in the deep thick coal seam.

## Engineering background

**Fig. 1:**
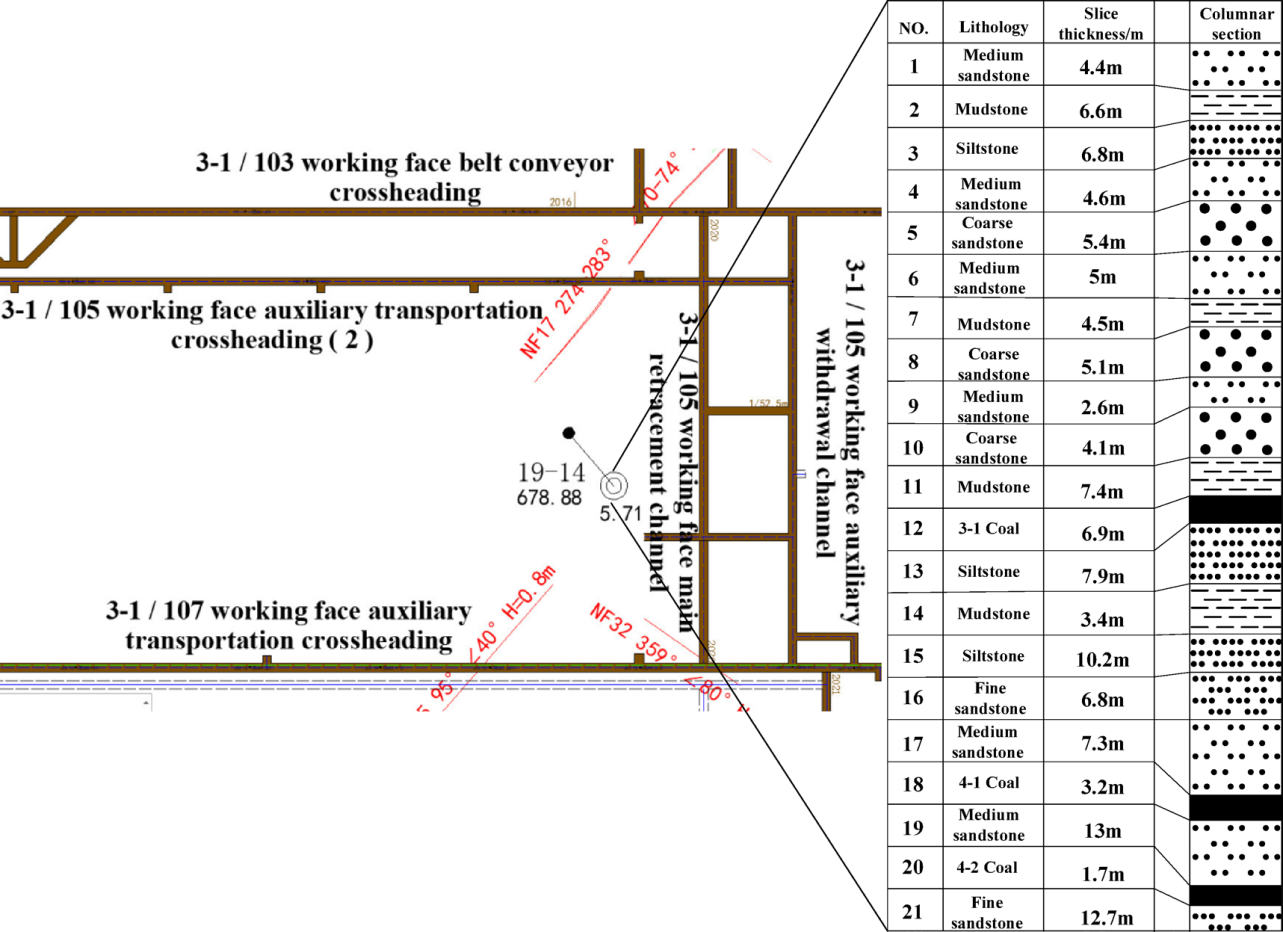
Composite borehole histogram.

The Dongsheng Coalfield mine is located in Yijinholo Banner, Ordos City, Inner Mongolia Autonomous Region, and covers an area of 4044 km^2^. The mine has main and secondary vertical shafts and 2 wind shafts, with a total of 4 shafts, and a production capacity of 15 million t/a with a service life of 103.3 years. The coal seams in the field are at a depth of 560 ~1000 m, with the 3-1 coal seam at a depth of 560,840 m, having an average thickness of 6.9 m, and a dip angle of about 1°, making it nearly horizontal. The overburden of the coal seam is stable throughout the area. The 4-1 coal seam is located about 46 m below the lower part of the 3-1 coal seam, with an average thickness of 3.2 m. The 4-2 coal seam is located about 13 m below the lower part of the 4 -1 coal seam, with an average thickness of 1.7 m. The 4-1 and 4-2 coal seams are distributed throughout the area, and the comprehensive column diagram of the mine is shown in Fig. 1. The 4-1 and 4-2 coal seams are stable in terms of position and thickness, and there is no danger of impact.

According to the coal rock tendency identification standard, the 3-1 coal seam exhibits a strong impact tendency, while the top and bottom slabs have a weak impact tendency. A microseismic event with an energy of 2.1 × 10^6^J and a microseismic magnitude of 2.3 was detected by the Coal Mine Microseismic Monitoring System (CMMMS) on 2 April 2021. Another microseismic event with the same energy and magnitude occurred in the working face of 3-1105 as measured by the ARAMIS microseismic monitoring system on 11 June 2021. Additionally, a microseismic event with an energy of 7 × 10^6^J occurred in the working face of 3-1105 as measured by the ARAMIS microseismic monitoring system. Due to the relatively strong dynamic events during the mining of the 3-1 coal seam, it is proposed to mine the 4-1 and 4-2 coals as the protective seams, and the 3-1 as the protected seam, and to analyze the unloading effect of the No. 4 coal protective seams (group) on the 3-1 coal seam and its surrounding rocks. The protective seams (group) of the No.4 coal define the 4-1 coal as the upper protective layer and the 4-2 coal as the lower protective layer.

## Feasibility analysis of upstream mining with double protection layer

In the process of mining the protective layer of 3-1 coal seam with 4-1 and 4-2 as the protective layer, it is necessary to study whether the 3-1 coal seam can continue to be mined when the 4-1 and 4-2 coal seam mining is completed. Under normal circumstances, the mining of the upper coal seam will generally not have a significant impact on the lower coal seam when the upper coal seam is mined first and then the lower coal seam is mined. However, in the case of upward mining, mining the lower coal seam first generally affects and damages the upper coal seam. In severe cases, it may render the upper seam unmineable. Hence, it is essential to evaluate the recoverability of the 3-1 coal after the protective seams are mined.

The main methods to judge whether 3-1 coal can continue mining are comprehensive ratio value criterion, three-zone discriminant method, surrounding rock balance method and so on. This paper mainly uses the comprehensive ratio discriminant method to calculate.

The comprehensive ratio discrimination method utilizes the mining influence coefficient, denoted as ratio *K* representing the distance between upper and lower coal seams and the thickness of the lower coal seams, to evaluate the viability of upward mining based on the *K* value. The formula for the mining influence coefficient is presented in;

1 $$K{\text{=}}\frac{{\text{1}}}{{\frac{1}{{{K_1}}}+\frac{1}{{{K_2}}}+\cdots +\frac{1}{{{K_{\text{n}}}}}}}$$

Where $${K_{\text{1}}}{\text{=}}\frac{{{H_1}}}{{{M_2}}}$$, $${K_{\text{2}}}{\text{=}}\frac{{{H_{\text{2}}}}}{{{M_{\text{3}}}}}$$, ···, $${K_{\text{n}}}{\text{=}}\frac{{{H_{\text{n}}}}}{{{M_{{\text{n+1}}}}}}$$

*H*_1_, *H*_2_, ···, *H*n are the vertical distances from coal seams m_2_, m_3_, ···, m_*n*+1_ to m_1_,m.

*M*_2_, *M*_3_, ···, *M*_*n*+1_ are respectively the mining heights of the lower coal seams, m.

According to the practice of up and down mining and research results in China, upward mining can be carried out normally when the integrated ratio *K*>6.3^39^.

Firstly, the recoverability of 3-1 coal is discriminated, according to the comprehensive bar chart of the mine, the vertical distance *H*_1_ from 4-1 coal to 3-1 coal is 35.6 m, the vertical distance *H*_2_ from 4-2 coal to 3-1 coal is 47.3 m, and the mining heights *M*_2_ and *M*_3_ of 4-1 and 4-2 coals are 3.5 m and 1.75 m, respectively, and substituting the calculations, it is:

$$K{\text{=}}\frac{{\text{1}}}{{\frac{1}{{{K_1}}}+\frac{1}{{{K_2}}}}} \approx {\text{7}}{\text{.45}}$$

At this time, the *K* value is more than 6.3 as the normal upward mining interval, and the 3-1 coal seam can be mined normally.

Secondly, the same method is used to judge the recoverability of 4-1 coal. Compared with 3-1 coal, only one coal seam is mined in the lower part of 4-1 coal, so the judgment method is different. When *K* > 7.5^39^, it is considered that mining 4-2 coal first has no effect on 4-1. The vertical distance *H*_3_ from 4-2 coal to 4-1 coal is 13 m, and the mining height *M*_1_ of 4-2 coal is 1.7 m.

$$K{\text{=}}\frac{{\text{1}}}{{\frac{1}{{{K_{\text{3}}}}}}} \approx {\text{7}}{\text{.6}}$$

At this time, the *K* value is greater than 7.5 for the normal upward mining interval, and the 4-1 coal seam can be mined normally.

According to the results obtained by the ratio value criterion, the 4-2 and 4-1 coal seams are first mined as the protective layer of the 3-1 coal seam, and then the mining scheme of the 3-1 coal is feasible.

## Numerical simulation analysis of unloading effects in double protection layer mining

### Numerical modelling

The establishment of numerical model should reflect the real mining situation of the project site as much as possible. Taking the A05 working face of the third mining area of the mine as an example, the FLAC3D numerical model is established. The model contains 156,800 units and 169,050 nodes, and the length, width and height of the model are 800 m × 300 m × 250 m respectively. Due to the actual stratigraphic inclination of 1–3°, the model depth is set as a horizontal stratum for the convenience of calculation in FIAC3D. The model burial depth is 600 m, and a self-gravitational stress of 14.7Mpa is applied to the upper boundary, with the Mohr-Coulomb yield criterion being adopted.The physical parameters of each rock layer in the numerical model are based on data measured before mining, as shown in Table 1.

**Fig. 2:**
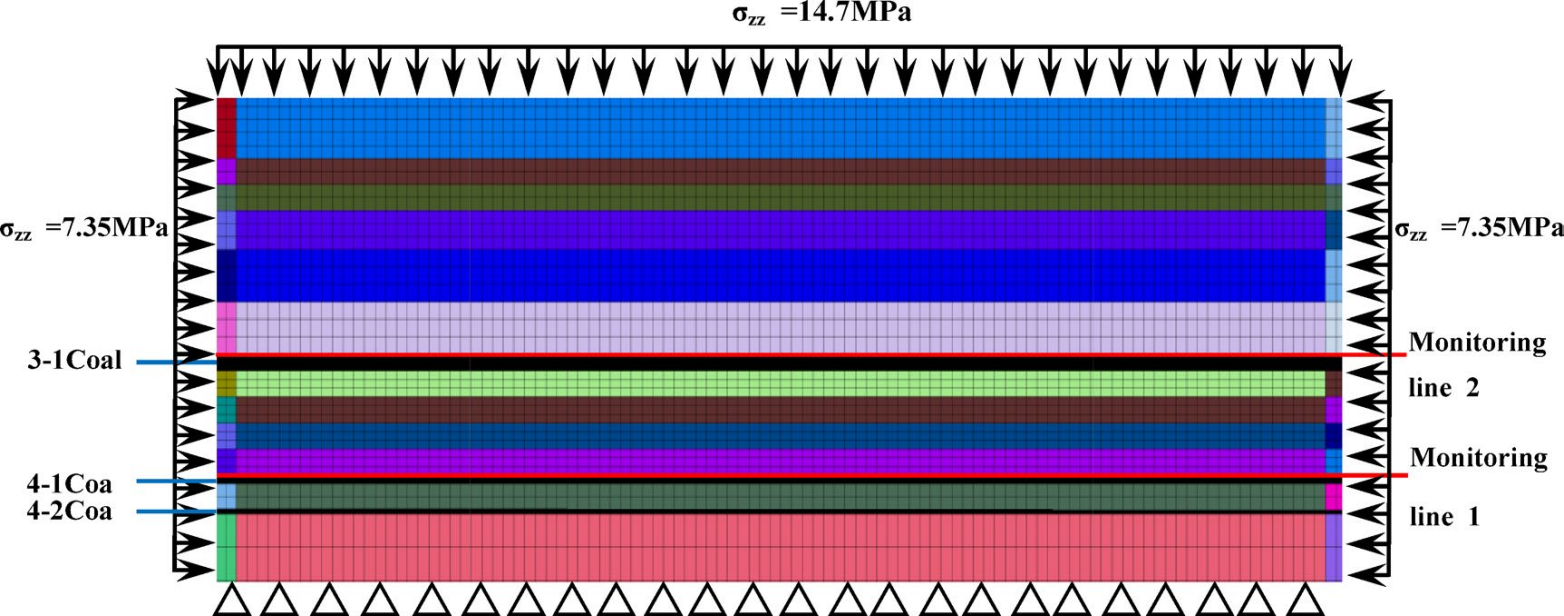
Numerical model and sketch map of survey line position.

The advancing distance of the 4-2 coal seam is simulated at 100 m, 200 m, 300 m, 400 m, and 500 m, with the open area being modeled using a Double-Yield model. During the mining of the 4-2 coal seam, the 4-1 coal seam is designated as the protected layer, and the maximum principal stress in the 4-1 coal seam is monitored. To facilitate comparison and analysis with the stress evolution of the 3-1 coal seam during the mining of the No.4 protected seam (group), two monitoring lines were set up at the coal-rock interface of the roof of the 4-1 coal seam and the 3-1 coal seam, respectively. The numerical model and monitoring line settings are depicted in Fig. 2.

**Table 1:** Physical and mechanical parameters of the coal seam.

	Bulk (Gpa)	Shear (Gpa)	Friction(°)	Cohesion (Mpa)	Tension (Mpa)	Poisson
Medium sandstone	13.3	2.5	37	4	1.2	0.2
Mudstone	7.1	5.1	25	1.2	1.1	0.2
Medium to fine sandstone	12.3	8.3	35	3.4	3.2	0.23
Fine sandstone	8.4	5.9	27	1.6	1.7	0.14
Coarse sandstone	26.4	20.7	34	4.3	3.8	0.22
Coal	4.8	1.2	27	1	0.8	0.31
Siltstone	15.2	9.4	30	2.8	2.4	0.2

## Model calculation results

### Mining analyses of the lower protective layer

**Fig. 3:**
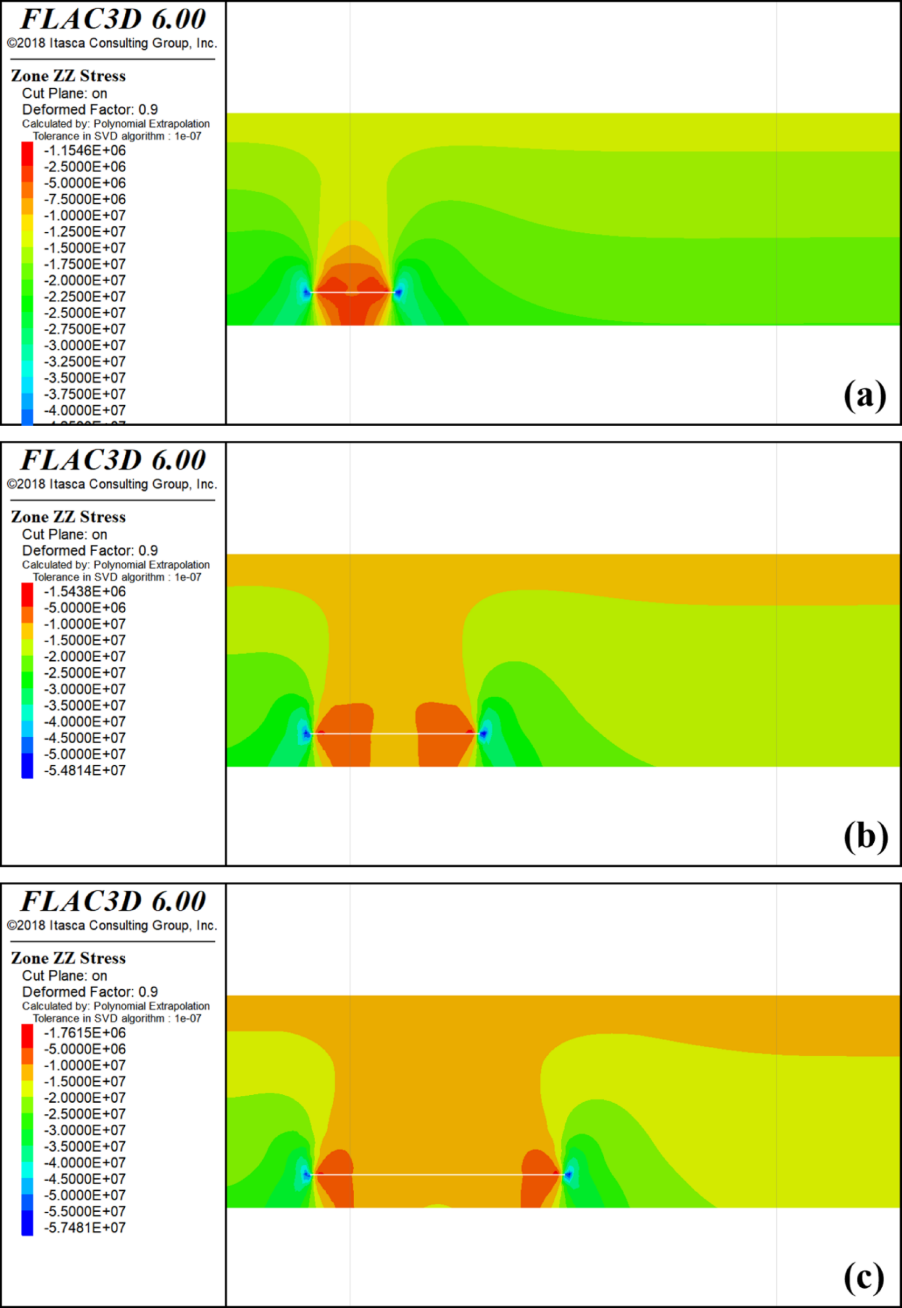

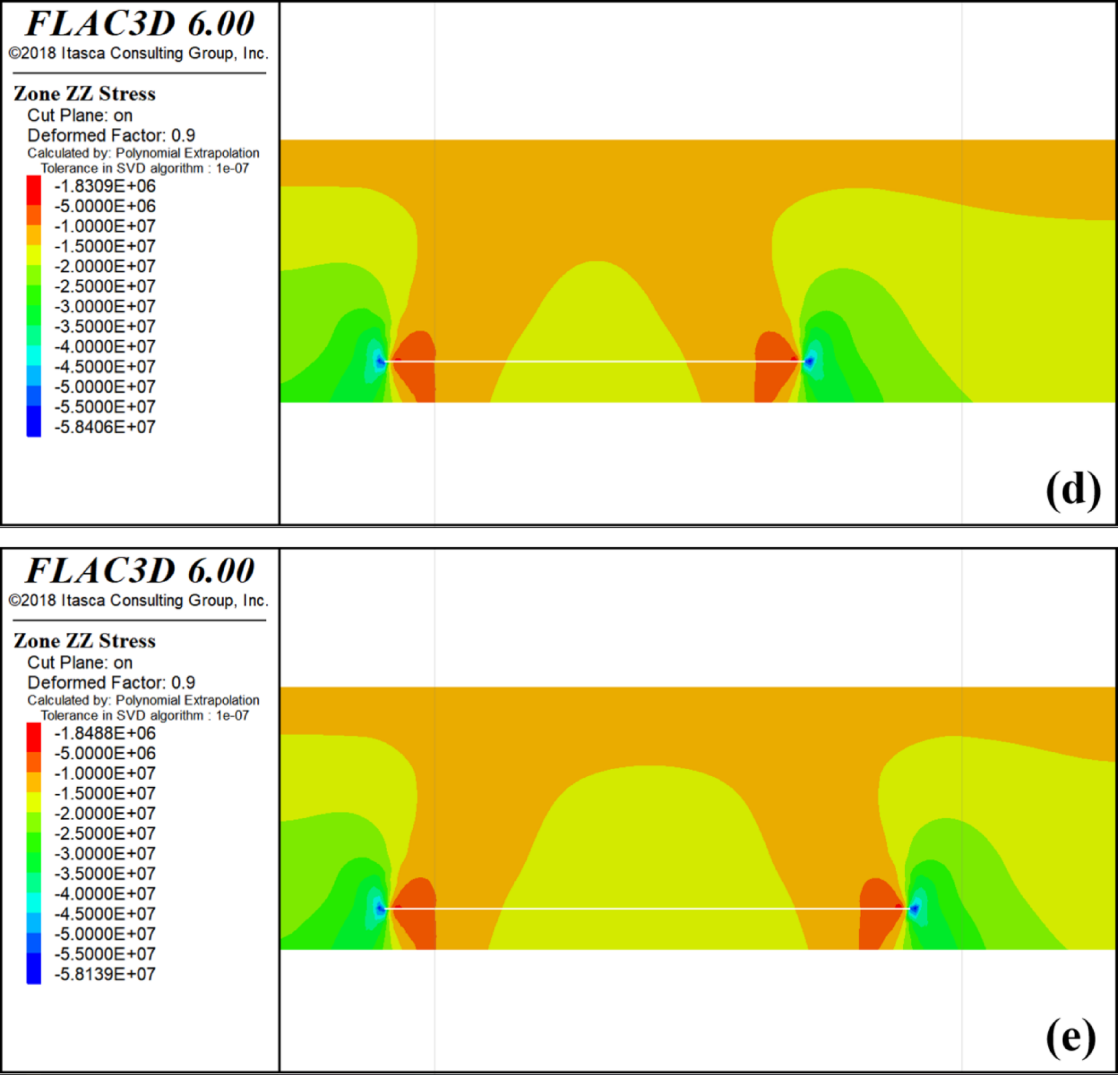
Mining 4-2 coal stress distribution cloud map. **a** Mining 100 m Stress Cloud; **b** Mining 200 m Stress Cloud; **c** Mining 300 m Stress Cloud; **d** Mining 400 m Stress Cloud; **e** Mining 500 m Stress Cloud.

Throughout the protective layer mining process, extraction of the lower protective layer causes the overlying rock layer to transition from a state of triaxial compression to pressure relief. Consequently, this redistribution of stress within the overlying rock layer transfers stress from the upper part of the coal seam to the sides, resulting in the formation of stress concentration zones in the surrounding rocks on both sides. Please refer to Fig. 3 for an illustration of the vertical stress distribution during the mining of 4-2 coal.

**Fig. 4:**
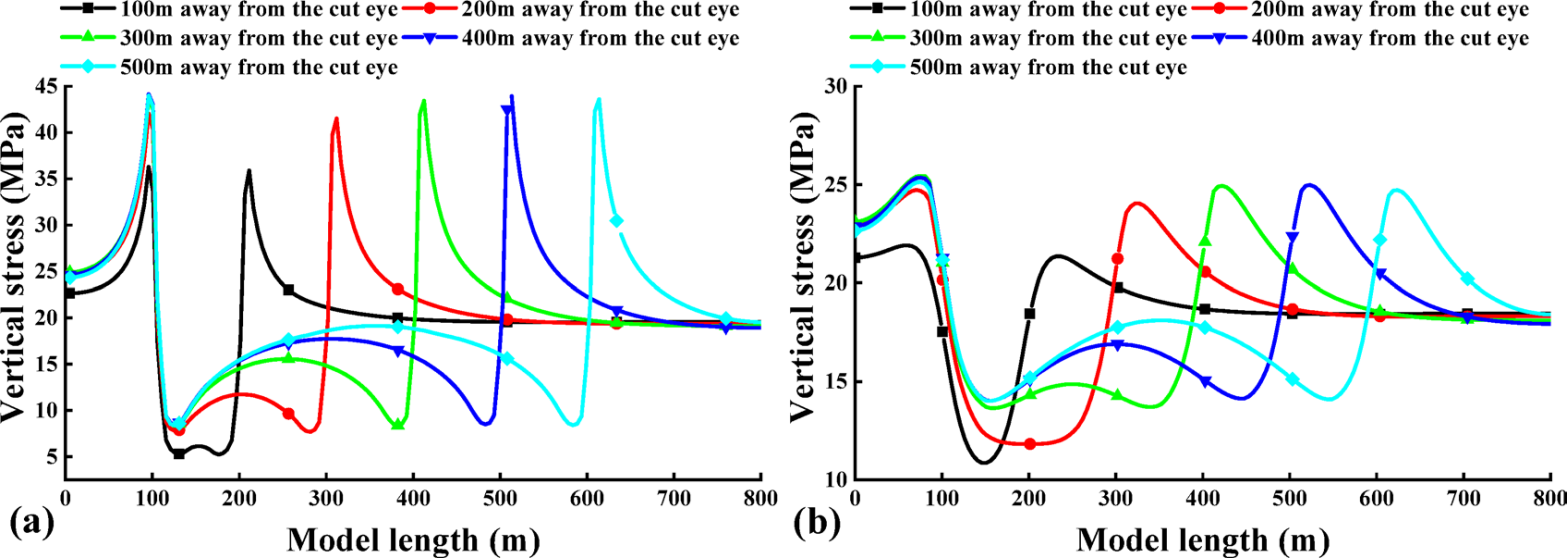
The vertical stress distribution map of coal seam during excavation of 4-2 coal seam. **a** 4-1 coal; **b** 3-1 coal.

The curves in Fig. 4. show the evolution of the vertical stresses in the 4-1 coal and the 3-1 coal along the advancing direction of the working face. The figure illustrates the distribution characteristics of vertical stress for 4-1 coal and 3-1 coal as the 4-2 coal face advances. The horizontal axis represents the length of the model, while the vertical axis represents the vertical stress of 4-1 coal and 3-1 coal. The original rock stress of 4-1 coal and 3-1 coal within the protected layer is approximately 18.5–19.7 MPa.

When the 4-2 coal seam advances by 100 m, the vertical stress on the 4-1 and 3-1 coal seams above the starting cut of the protected seam and the mining hollow area is approximately 17.4 MPa, while the vertical stress on the 3-1 coal seam is 17.5 MPa. The stress distribution in the vertical direction near the starting cut of the 4-1 and 3-1 coal seams exhibits minimal changes compared to the original rock stress. The stress concentration is observed in the peripheral rock to the left of the starting cut and the right of the stopping line, which are critical areas for the protected seam. In the upper part of the mining area, there is a slight reduction in stress compared to the original rock stress, indicating limited mining-induced movements and influences on the protected layer.

Upon advancing 200 m, the vertical stress of 4-1 coal ranges from 7.68 to 13.6 MPa, whereas the vertical stress of 3-1 coal ranges from 11.8 to 18.3 MPa. Initially, the effect of decompression is observed over a gradually expanding range, with the stress concentration area still located in the peripheral rock on the left side of the starting cut and the peripheral rock on the right side of the stop mining line.The degree of overlying coal rock decompression increases with the deepening of mining in 4-2 coal. When the mining depth reaches 500 m, the maximum vertical stress of 4-1 coal reaches 44 MPa, appearing above the 4-2 coal starting cut. As it passes through the stress concentration area above the starting cut, the vertical stress of 4-1 coal decreases significantly. The maximum vertical stress of 3-1 coal is 25 MPa, also appearing above the 4-2 coal starting cut. The stress curve of 4-1 coal at this point forms a ‘saddle’ shape, with high vertical stress distribution on both sides and lower in the middle.

With a limited advancement distance of the protected layer’s working face, the degree of decompression and its influence range remain minimal. As mining progresses further, the unloading zone above goaf expands. When the pressure relief range reaches a certain scale, due to the movement of the overlying strata, a part of the protective layer is compacted, so that the degree of pressure relief in the protective layer is weakened, so that the pressure is increased and finally stabilized at a certain point.

### Determination of upper protective layer starting cut

**Fig. 5:**
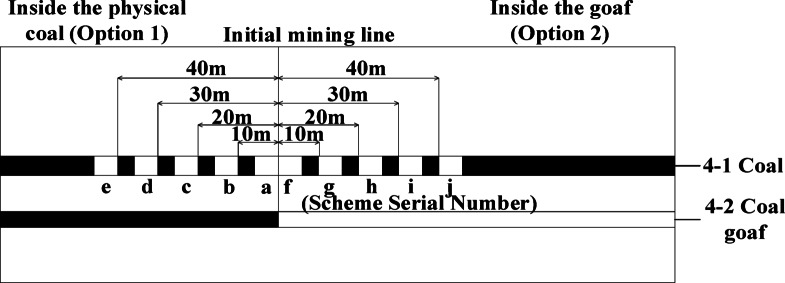
Schematic diagram of the determination of the starting cut scheme.

Upon completing the simulation of 4-2 mining, the next step involves determining the location of the starting cut for mining the 4-1 coal seam. When selecting the starting cut location, initially identify the general area, which can be categorized into two options: Scheme 1 places the starting cut within the solid coal region of the 4-2 coal seam, while Scheme 2 locates it in the upper section of the 4-2 coal seam’s mining airspace. Refer to Fig. 5. for the schematic representation of the starting cut selection process.

**Fig. 6:**
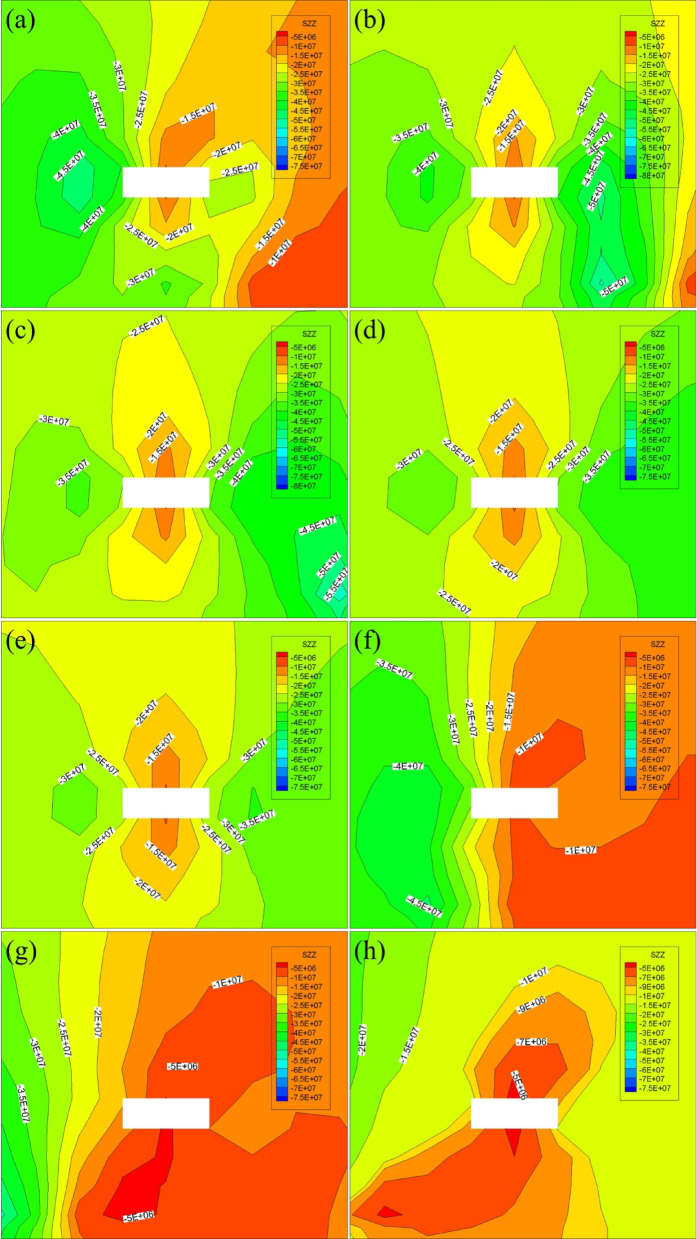

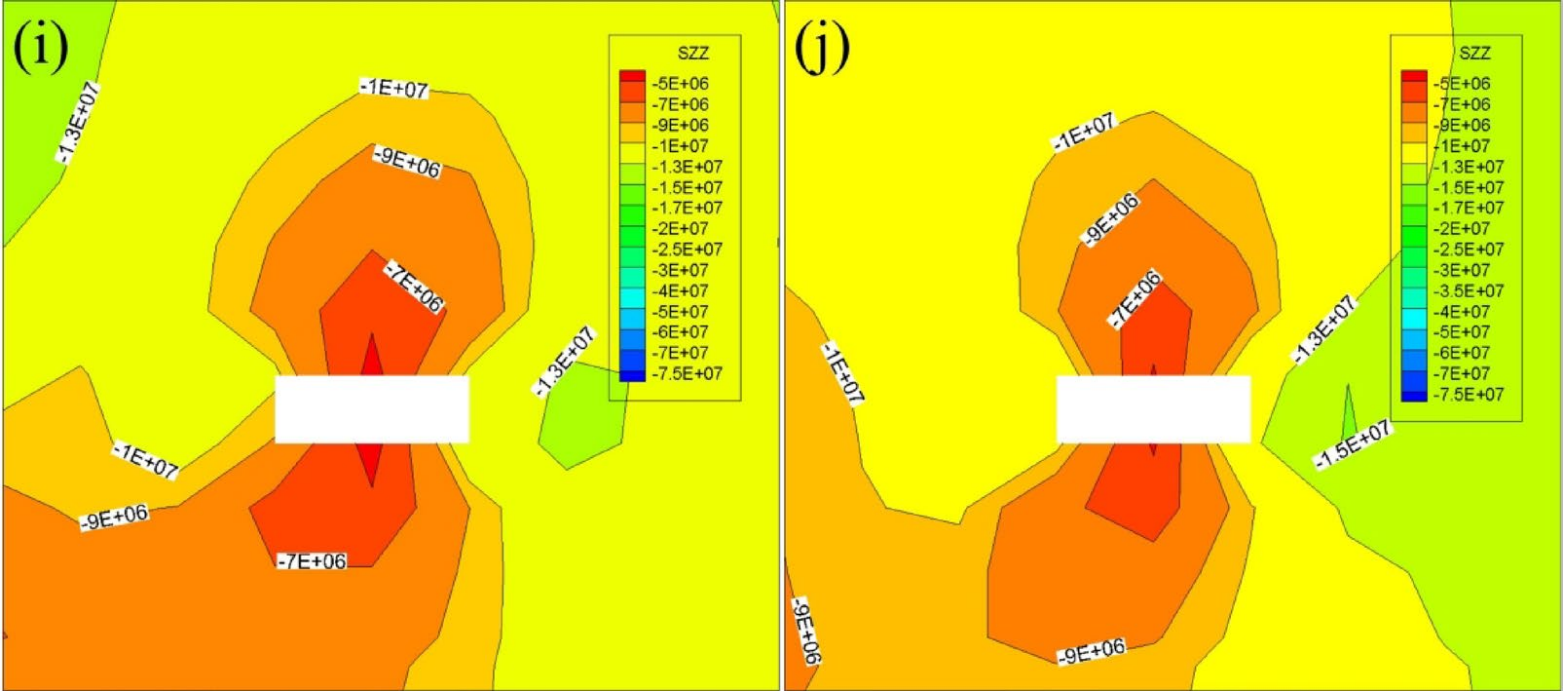
Stress distribution map of the surrounding rock in the starting cut. **a** Contour map of stress distribution for option a; **b** contour map of stress distribution for option b; **c** contour map of stress distribution for option c; **d** contour map of stress distribution for option d; **e** contour map of stress distribution for option e; **f** contour map of stress distribution for option f; **g** contour map of stress distribution for option g; **h** contour map of stress distribution for option h.

According to the schematic diagram of the starting cut scheme, FLAC3D numerical simulation is carried out on the starting cut position of 4-1. The distance from the starting cut to the initial mining line is used to divide the scheme into five different schemes, named according to the distance from the starting cut to the initial mining line 0 m, 10 m, 20 m, 30 m, 40 m were named as scheme a, scheme b, scheme c, scheme d, scheme e; Similarly, the starting cut in Scheme 2 is named as scheme f, scheme g, scheme h, scheme i and scheme j according to the distance of 0 m, 10 m, 20 m, 30 m and 40 m from the initial mining line. According to the contour map of stress distribution, the stress situation of starting cut is analyzed to find out the optimal scheme of starting cut selection. Figure 6 shows the position and stress distribution map of starting cut.

The arrangement of all the starting cut in Scheme 1 is depicted in Fig. 6. The upper and lower parts of the starting cut exhibit dense contours. The stress escalates from approximately 12 MPa to about 23 MPa over a short distance. The stress level in the left and right sections of the starting cut averages around 30 MPa. The surrounding rock stress experiences a significant increase, making roadway maintenance challenging. In Scheme 2, the stress in the upper section of the starting cut is below 10 MPa, indicating a more favorable arrangement compared to Scheme 1. Considering safety aspects, the layout of the starting cut in Scheme 2 is recommended.

The arrangement position of scheme f in Scheme 2 is directly above the 4-2 coal starting cut, the left side of the starting cut is the stress concentration area, the stress of the left gang is obviously larger than that of the right gang, the maximum stress is about 30 MPa, and the vertical stress of the roof is about 8 MPa; the position of the starting cut in scheme g is located in the 10 m after the position of the scheme f, and the contour plot of the stress of the left gang of the scheme g starting cut is very dense from the figure, which indicates that the stress of the left gang of the starting cut rises significantly, and the roadway maintenance and maintenance of the roadway are very important. It shows that the stress of the left gang of the starting cut rises significantly, and it is difficult to maintain the roadway, and the vertical stress of the roof is about 6 MPa; the location of the starting cut of scheme h is located at 20 m after the starting line, and the stress of the two gangs of the starting cut is about 12 MPa, and the vertical stress of the roof is about 6 MPa, and the stress of the left gang changes less in a short distance compared with that of the scheme g; the location of the starting cut of scheme i is located at 30 m after the starting line, and the stresses of the two gangs are about 11 MPa, the left and right gangs do not have obvious area of rapid stress rise, the maximum vertical stress of the roof is 5 MPa; the location of the starting cut of scheme j is located in the back 40 m of the start line, the stress of the left and right gangs is about 10 MPa, the stress rises slowly, the left gang of the starting cut is easier to maintain compared with scheme h, i, and the minimum vertical stress of the roof is about 5 MPa. Compared with the previous scenarios, scheme j has less surrounding rock stress and a more uniform contour distribution.

The stress in the surrounding rock of scheme j is lower compared to the previous scheme, and the contour distribution is more evenly spread. Based on this observation, scheme j is selected as the location for the 4-1 coal starting cut, positioned 40 m away from the side of the mining area directly above the 4-2 coal starting cut.

### Mining analysis of the upper protective layer

**Fig. 7:**
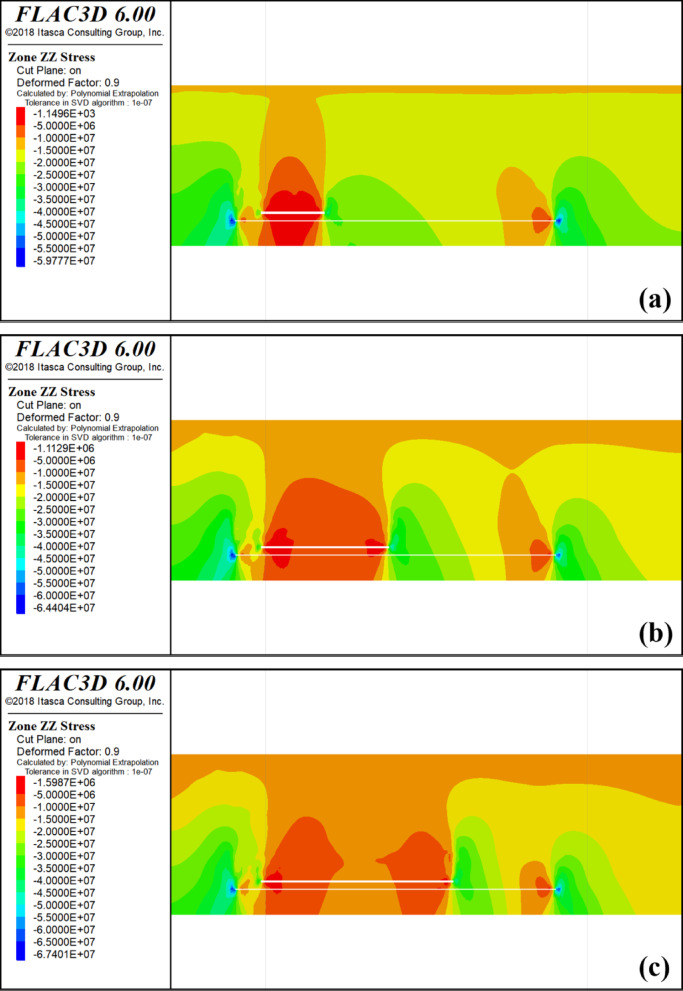

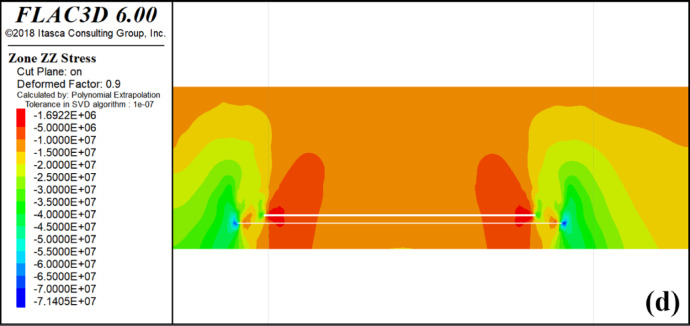
Mining 4-1 coal stress distribution cloud map. **a** Mining 100 m Stress Cloud; **b** Mining 200 m Stress Cloud; **c** Mining 300 m Stress Cloud; **d** Mining 400 m Stress Cloud.

After determining the location of the 4-1 coal starting cut, a simulation of the mining of the 4-1 coal seam was conducted following the completion of the 4-2 mining. This simulation aimed to analyze the stress changes and unloading behavior within the 3-1 coal seam when it served as the protected seam during the mining of the lower double-protected seam.The simulated mining of the 4-1 coal seam was divided into four phases, each representing 100 m, 200 m, 300 m, and 400 m, respectively. The cloud diagram depicting the vertical stress distribution during the mining of the 4-1 coal seam is illustrated in Fig. 7.

**Fig. 8:**
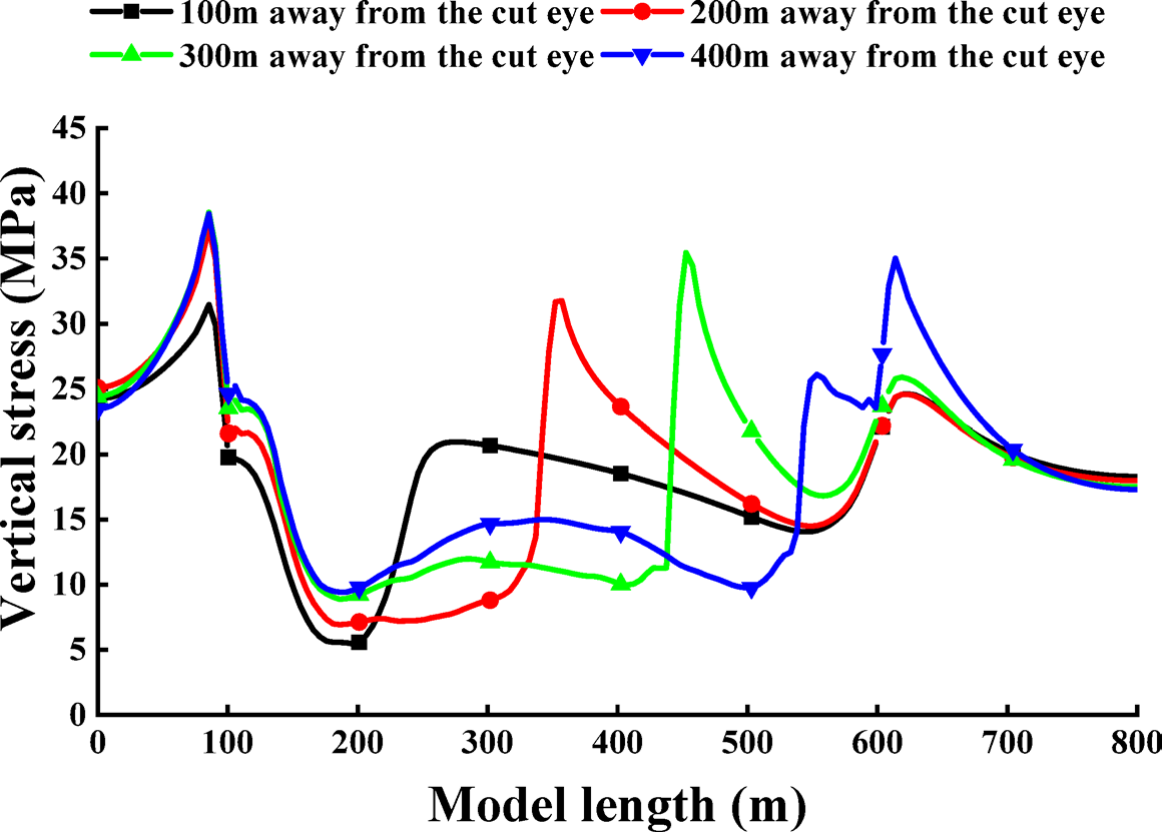
Vertical stress distribution in the direction of inclination of coal 3-1 after excavation of coals 4-2 and 4-1.

According to the simulation results, the open-off cut of 4-1 coal is determined as scheme j, and the above diagram (Fig. 8) is the vertical stress change curve in the dip direction of 3-1 coal when the open-off cut at scheme j continues to excavate 4-1 coal.

During the mining of the 4-1 coal seam, the stress distribution curve along the trend direction of the 3-1 coal seam is analyzed. Within the initial 200 m of the model, the vertical stress curve of the 3-1 coal seam exhibits a pattern of initial increase, followed by rapid decrease, slight increase, and subsequent decrease. This behavior is attributed to the presence of the lower protective layer, where the vertical stress of the 3-1 coal seam is influenced by both the upper and lower protective layers during mining activities in the upper protective layer. The initial increase in vertical stress within the first 100 m is a result of the superposition of stress concentration from the lower protective layer and the stress induced by mining activities in the upper protective layer, leading to an overall elevation of vertical stress levels. At the 100 m mark of the model, the lower protective layer, namely the 4-2 coal seam, was extracted as a protective measure, resulting in a significant reduction in the vertical stress of the 3-1 coal seam. At this time, the upper protective layer, namely 4-1 coal, has not been mined as a protective layer. The mining position of 4-1 coal is located at 140 m of the model, and the stress concentration phenomenon is only reduced but not eliminated, so there will be a slight increase. After the upper protective layer begins to be mined at 140 m of the model, the vertical stress of 3-1 coal decreases rapidly. The coal seam gradually relieves pressure.

The minimum vertical stress of the 3-1 coal seam between 200 m and 500 m in the model exhibits a gradual increase due to the ongoing extraction of the upper protective layer, specifically the 4-1 coal seam. This phenomenon can be attributed to the continuous compaction of a section within the upper protective layer during the mining of the 4-1 coal seam, resulting in a progressive rise in the minimum stress levels.

At the 500–800 m interval in the model, the vertical stress curve of the 3-1 coal seam exhibits a pattern of initial increase followed by decrease, reaching its minimum at 600 m before rising and falling again. The initial increase observed at 500 m is attributed to the presence of the stopping line of the upper protective layer at 540 m, where the right side experiences stress concentration, leading to an elevation in the vertical stress of the 3-1 coal seam.

At 580–600 m of the model, the vertical stress of 3-1 coal has decreased because the stop line of the lower protective layer is located at 600 m, while the stress concentration area of the upper protective layer is at 545–580 m. Therefore, at 580–600 m of the model, the stress concentration area of the upper protective layer has less influence on the vertical stress of 3-1 coal, and the pressure relief effect of the lower protective layer makes the stress of 3-1 coal show a downward trend at 580–600 m. Notably, At 600 m of the model, that is, the stop line position of the lower protective layer, the vertical stress of 3-1 coal shows an upward trend because the stress concentration area of the lower protective layer and the stress caused by the mining of the upper protective layer are superimposed to make the vertical stress of 3-1 coal rise. After 650 m of the model, the vertical stress curve of 3-1 coal decreases because the stress concentration area of the lower protective layer has passed and the stress decreases.

In Fig. 8, the segment between 200 m and 500 m within the model is identified as the region where the 3-1 coal seam experiences the most significant pressure relief, exhibiting symmetrical characteristics on both sides. The central area of the 3-1 coal seam represents a stable pressure relief zone, with a length of approximately 340 m along the strike length.

### Comparative analysis of the pressure relief effect of the protected layer

The evaluation of pressure relief indices for the extraction of the upper protective layer can be conducted by utilizing the stress release rate as a metric. The stress release rate (denoted as R) is calculated using the formula as:

2 $$R{\text{=}}\frac{{\sigma - \sigma ^{\prime}}}{\sigma } \times {\text{100\% }}$$

σ—Primary rock stress, MPa;

σ´—Is the post-mining stress, MPa。.

After mining, if the stress is lower than the original rock stress, resulting in a positive stress release rate, it indicates that the rock mass has been depressurized. Conversely, a negative stress release rate suggests stress concentration within the rock mass. Therefore, a higher stress release rate value signifies a greater degree of depressurization in the protected layer. During the mining of the protected seam, the deformation of the coal strata is primarily influenced by the stress release rate, which reflects the decompression rate of the coal bed rock layer. Thus, the stress release rate serves as a criterion for determining the full decompression of the coal bed rock layer. Based on the literature cited in this study, a decompression rate of 10% indicates complete decompression of the coal bed^40^.

**Fig. 9:**
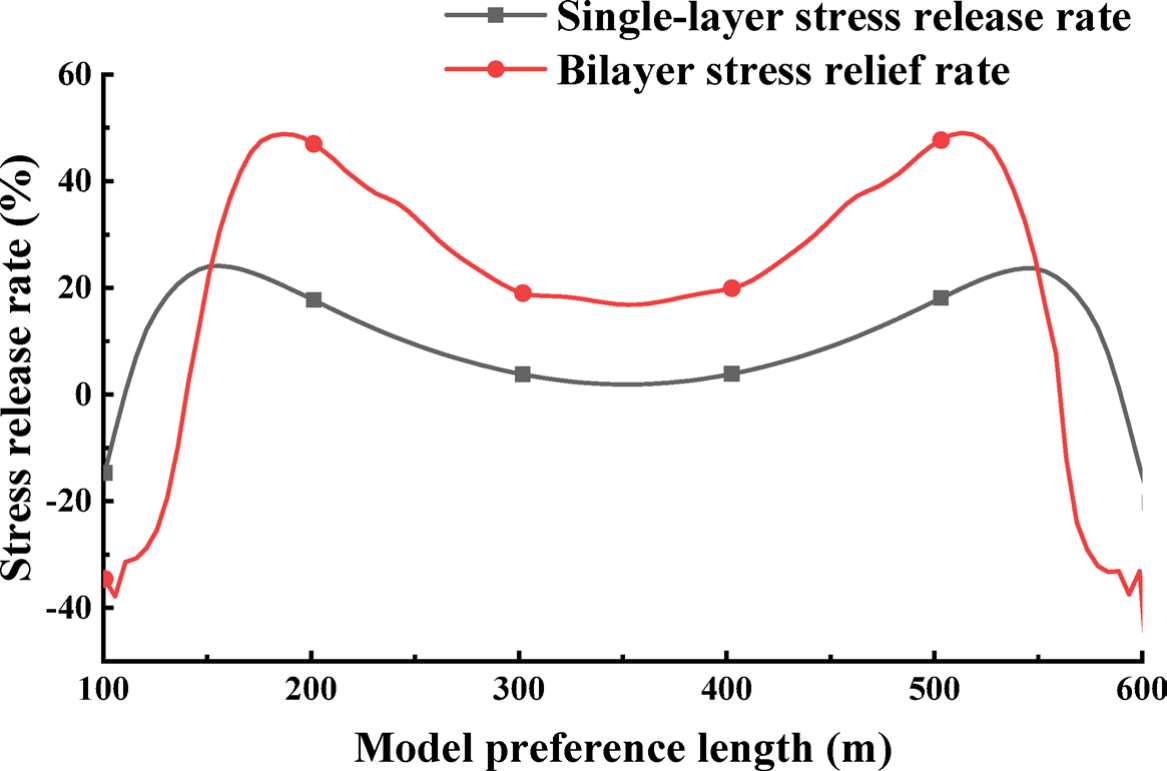
Comparison of stress release rate between single protective layer and double protective layer 3-1 coal.

The comparison curve diagram of the stress release rate of the 3-1 coal in single protection layer and double protection layer, based on the vertical stress data obtained from numerical simulation, is presented in Fig. 9. The stress release rate of the 3-1 coal is found to be negative in the first 15 m when mining the single protection layer, and negative in the first 35 m when mining the double protection layer. Additionally, the stress concentration area expands inward by 20 m compared to mining the single protection layer. The maximum stress release rate of the 3-1 coal is located at 150 m when mining the single protection layer, with a maximum stress release rate of 24%. In contrast, the maximum stress release rate is 48% and located at 180 m of the 3-1 coal seam when mining the double protection layer, moving inward by 30 m compared to the single protection layer, resulting in a 100% increase in stress release rate.

Upon analyzing the decompression effect, it is observed that when mining the single protective layer, only the intervals of 120–240 m and 450–570 m achieve a stress release rate of more than 10%, with only the 240 m interval achieving a more adequate decompression. In comparison, when mining the double protective layer, the stress release rate reaches negative values within the intervals of 130–140 m and 555–560 m, totaling 15 m with a decompression rate less than 10%. Furthermore, the intervals of 145–550 m achieve full pressure relief, with the minimum rate of pressure relief being 16%. This analysis suggests that the double protective layer can achieve a more ideal pressure relief effect compared to the single protective layer.

## Physical modelling experiment

### Physical modelling experiment procedures

After determining the location of the starting cut through numerical simulation calculations, a similar simulation test was conducted to study the development of off-seam fissures in the upper 3-1 coal seam and the surrounding overburden after the completion of the double-protected seam mining. The main equipment used for the test includes a plane stress similarity simulation experiment system and a Southern NTS-332R4 total station. The similarity simulation experiment system has a length, width, and height of 2 m, 0.2 m, and 1.5 m, respectively.

According to the test needs, the geometric similarity constant of the model is *C*_*l*_= 125, and the bulk density similarity constant is *C*_*γ*_= 1.5. Through the relevant formula^41–43^, combined with the actual situation on site and the size specifications of the two-dimensional test platform in the laboratory, the similar parameters of the test are finally determined as shown in the following Table 2.

**Table 2:** Similar parameters of the test model.

Similitude parameter					
*C*_*l*_	*C*_*γ*_	*C*_*t*_	*C*_*σc*_	*C*_*E*_	*C*_*µ*_
125	1.5	11	300	300	1

The main materials used in the similar simulation test are river sand, gypsum, calcium carbonate, and mica powder. The main filler of the model is river sand, and the binder is gypsum and calcium carbonate. When the ratio of river sand to cementing material is determined, the strength of the material can be adjusted by changing the ratio of calcium carbonate to gypsum. By analyzing the formation conditions of the mine and consulting the geological data and formation mechanical parameters of the study area, the proportion relationship between the total mass of similar materials and different filling materials in each layer is calculated by formula :

3 $$M = \gamma \cdot l \cdot b \cdot g \cdot q$$

In the formula :

*M*—the total mass of each layered similar material, kg;

*γ*—the bulk density of each layer of similar materials, *g/cm*^*3*^;

*l*—the length of the model, m;

*b*—the width of the model, m;

*g*—the thickness of each layer of the model, m;

*q*—material loss coefficient, *k* = 1.4.

According to the Mixing ratio^44^ of different coal lithology, the amount of material used in each layer of the test can be expressed as^45^:

4 $$\left\{ {\begin{array}{*{20}{l}}{{m_{\rm{h}}} = \frac{A}{{A + 1}} \cdot M}\\{{m_c} = \frac{1}{{A + 1}} \cdot \frac{B}{{B + C}} \cdot M}\\{{m_s} = \frac{1}{{A + 1}} \cdot \frac{C}{{B + C}} \cdot M}\\{{m_w} = \frac{1}{{10}} \cdot M}\end{array}} \right.$$

In the formula :

*m*_h_—Amount of river sand used;

*m*_c_—Amount of calcium carbonate;

*m*_s_—Amount of gypsum;

*m*_w_—Amount of water.

The ratio numbers A, B and C correspond to river sand, calcium carbonate and gypsum respectively. According to the ratio and bulk density of similar materials, the amount of similar materials in each layer is calculated, and the thickness of different strata and the mixing ratio of materials in the model are determined. The results are shown in Table 3.

**Table 3:** Analogous materials to model rock strata in the physical modelling experiment.

Serial number	Lithology	Model thicknes /cm	Mixing ratio	Capacityg /cm^3^	Sand /kg	Calcium Carbonate /kg	Gypsum /kg	Gross weight/kg	Water /kg	Number of layers
1	Coarse sandstone	3	7:7:3	1.6	23.52	2.35	1.01	26.88	2.69	1
2	Medium sandstone	2	7:7:3	1.6	15.68	1.57	0.67	17.92	1.79	1
3	Mudstone	2	8:6:4	1.5	14.93	1.12	0.75	16.80	1.68	1
4	Siltstone	10	7:5:5	1.6	78.40	5.60	5.60	89.60	8.96	5
5	4-2 Coal	1.5	8:6:4	1	7.47	0.555	0.375	8.40	0.84	1
6	Medium sandstone	10	7:7:3	1.6	78.4	7.85	3.35	89.5	8.95	5
7	4-1 Coal	2.8	8:6:4	1	13.944	1.036	0.7	15.68	1.568	1
8	Medium sandstone	6	7:7:3	1.6	47.04	4.71	2.01	53.76	5.37	3
9	Fine sandstone	6	7:8:2	1.6	47.04	5.38	1.34	53.76	5.24	3
10	Siltstone	6	7:5:5	1.6	47.04	3.36	3.36	53.76	5.38	2
11	Mudstone	2	8:6:4	1.5	14.93	1.12	0.75	16.80	1.68	1
12	3-1 Coal	3	8:6:4	1	14.94	1.11	0.75	16.80	1.68	1
13	Mudstone	6	8:6:4	1.5	44.79	3.36	2.25	50.4	1.68	3
14	Coarse sandstone	3	7:7:3	1.6	23.52	2.35	1.01	26.88	2.69	1
15	Medium sandstone	2	7:7:3	1.6	15.68	1.57	0.67	17.92	1.79	1
16	Coarse sandstone	3	7:7:3	1.6	23.52	2.35	1.01	26.88	2.69	1
17	Mudstone	4	8:6:4	1.5	29.87	2.24	1.49	33.60	3.36	2
18	Medium sandstone	3	7:7:3	1.6	23.52	2.35	1.01	26.88	2.69	1
19	Coarse sandstone	3	7:7:3	1.6	23.52	2.35	1.01	26.88	2.69	1
20	Medium sandstone	4	7:7:3	1.6	31.36	3.14	1.34	35.84	3.58	2
21	Siltstone	6	7:5:5	1.6	47.04	3.36	3.36	53.76	5.38	2
22	Mudstone	4	8:6:4	1.5	29.87	2.24	1.49	33.60	3.36	2
23	Medium sandstone	2	7:7:3	1.6	15.68	1.57	0.67	17.92	1.79	1

As the actual mining length of the coal seam in the field is too long, the plane stress similarity simulation experiment system cannot simulate the full length. Therefore, the actual length of the coal seam is scaled down, with the actual length being 2:1 compared to the test length. The ratio of similar materials is then determined based on the similarity constants and the actual conditions of the stratum. The upper part of the model is determined, as well as the pressure around the model, and the model is set up. The 4-1 and 4-2 coal seams are painted black with pigment, while the area of the 3-1 coal seam is indicated by a dotted line composed of black dots.

**Fig. 10:**
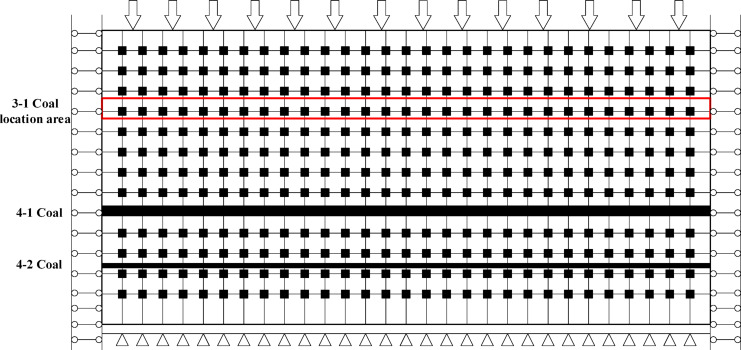
Schematic diagram of modeled measurement points and coal seam locations.

After modeling, the arrangement of reflectors and detection points was determined, and the specific locations are shown in Fig. 10. The vertical distance and horizontal distance interval of each measuring point located above the 4-1 coal is 5 cm, the vertical distance and horizontal distance interval of each measuring point below the 4-1 coal is also 5 cm, and the spacing between the upper and lower two lateral lines adjacent to the 4-1 coal is 10 cm. Stress monitoring devices were buried at the 3-1 coal seam, with a spacing of 25 cm between each stress monitoring device, and the distances of the stress boxes from the left end of the model were 25 cm, 50, 75, 100, 125, 150, and 175, respectively. Figure 10 shows the locations of reflectors and probe points in the model used to measure the deformation and displacement of the upper 3-1 seam overburden and the development of extra-seam fissures after the completion of double-protection mining.

### Results and analysis

**Fig. 11:**
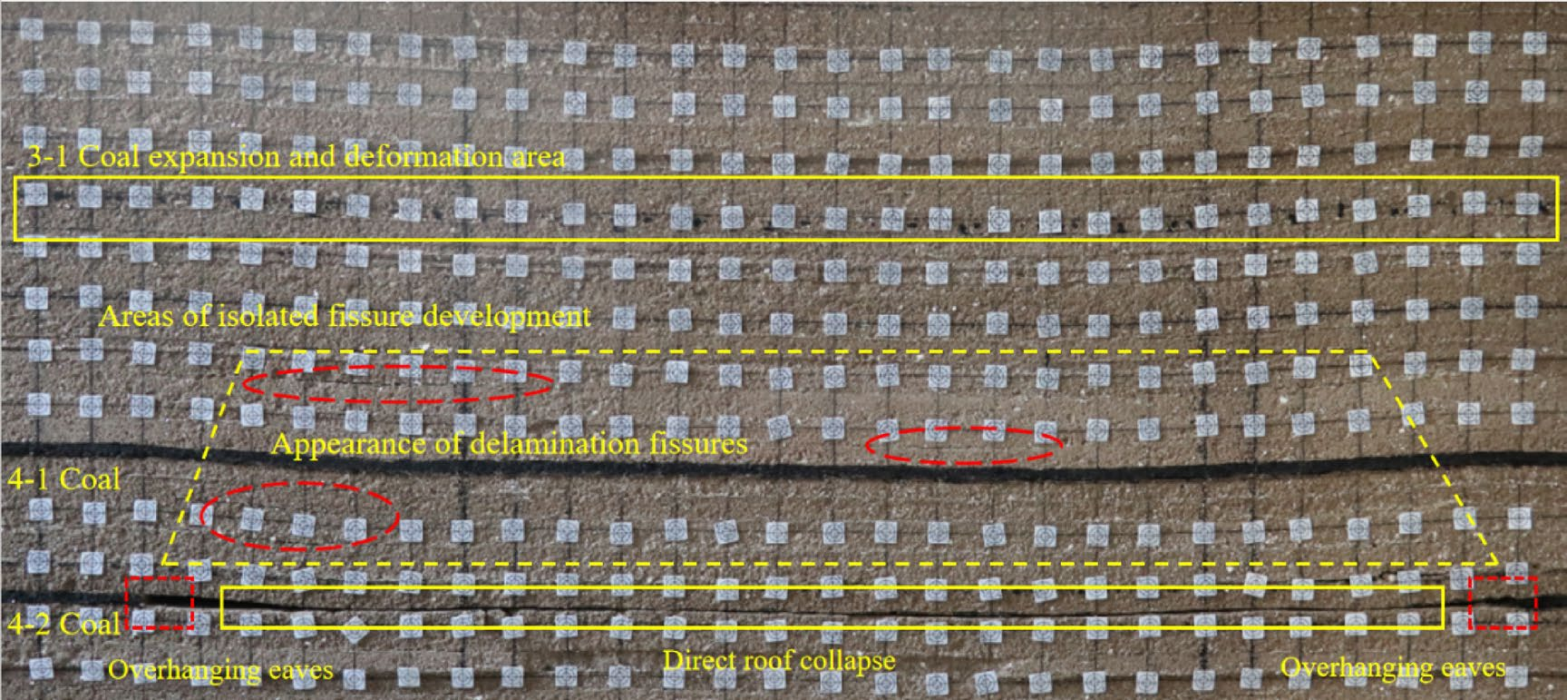
Fracture development of the 4-2 coal seam in mining.

During this similar simulation test, a mining scheme was determined through numerical simulation calculations, and a similar physical model was excavated. The 4-2 coal seam was mined first, and the final mining result is shown in Fig. 11. At 80 m of coal seam advancement, the fissure above the coal seam starts to develop. At 160 m, a large fissure in the overlying rock stratum of the 4-2 coal appears, causing obvious bending. As the mining time of the 4-2 coal increases, the fissure generated by the movement of the 4-2 coal roof gradually closes under the influence of self-weight. When the coal seam is advanced to 500 m, the overlying strata of 4-2 coal seam undergo obvious bending deformation, which compacts the middle of the goaf of 4-2 coal seam, and the development height of the fracture zone is about 26 m. At this time, the 4-1 coal seam above the 4-2 coal seam has obvious bending deformation and has a certain impact on the 3-1 coal seam mining area.

**Fig. 12:**
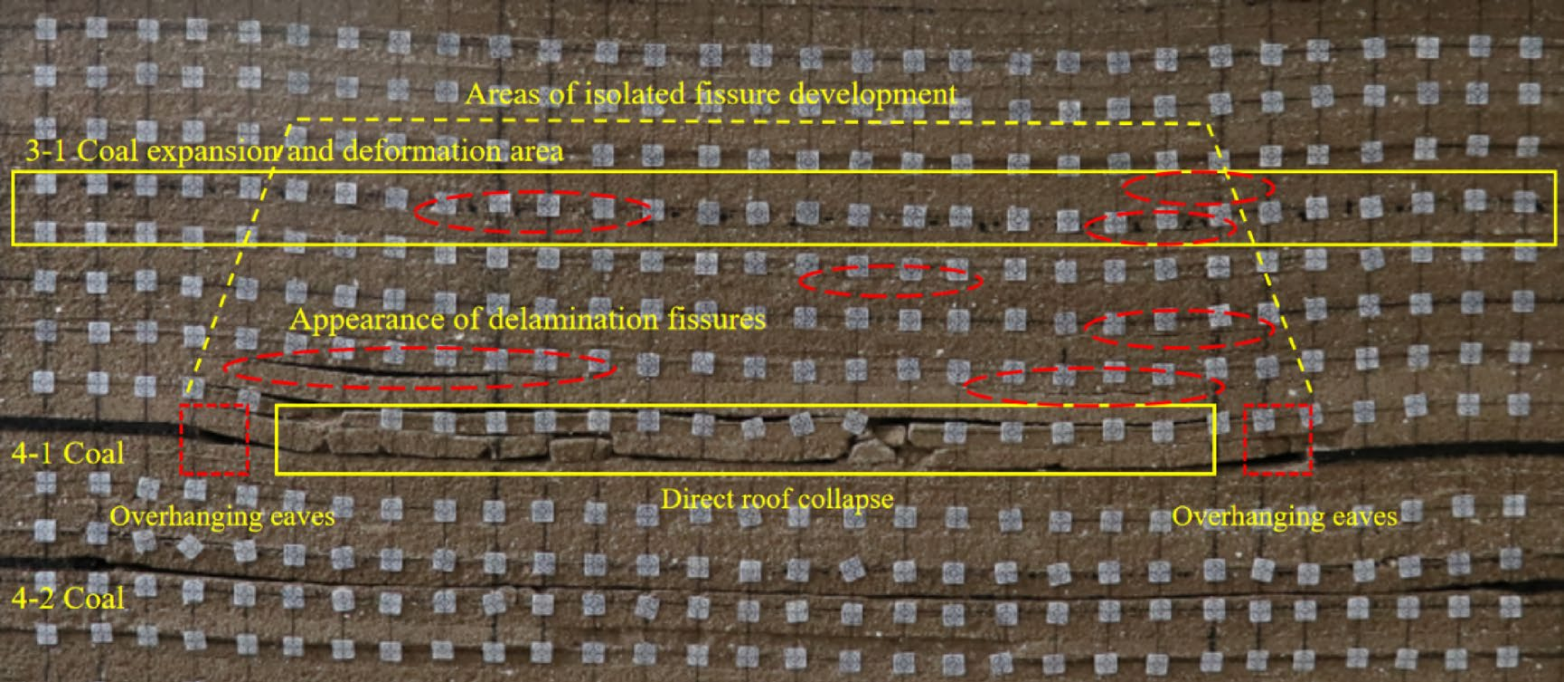
Mining 4-1, 4-2 coal seam fissure development map.

Following the extraction of the 4-2 coal seam, the 4-1 coal seam was subsequently mined. As a result of the 4-2 coal mining activities, significant fissures had already formed above the coal seam when the 4-1 coal was advanced 120 m, leading to partial collapse at the 200 m mark. Since then, the collapse of the overlying strata has occurred successively with the advancement of the working face. After the completion of the 4-1 coal mining, the fractured zone has developed to the area near the 3-1 coal seam, indicating that the 3-1 coal seam is under the mining influence of the underlying double protective layer. The height of the caving zone above the 4-1 coal seam is about 7 m, and the development height of the fractured zone is about 38 m. The final results of the excavation of 4-2 coal and 4-1 coal are shown in Fig. 12.

**Fig. 13:**
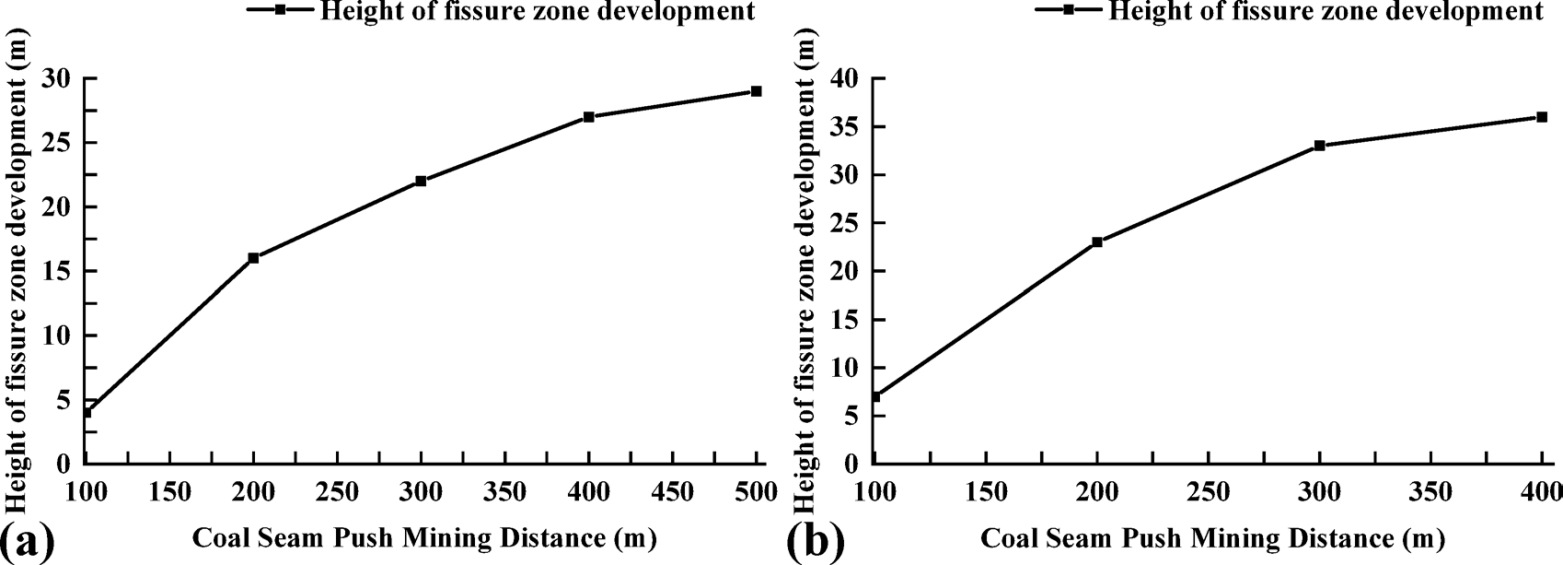
Fissure zone development curve diagram. **a** 4-2 Coal mining fissure development folding line map; **b** 4-1 Coal mining fissure development folding line map.

After the mining of 4-2 coal seam and 4-1 coal seam is completed, compared with only excavating 4-1 coal seam, the settlement of 3-1 coal seam location area is more obvious; the fracture development area of the separation layer has increased significantly; the bending deformation range and the number of separation layers at the 3-1 coal seam increase; the development curve of the fracture zone during the mining of 4-1 and 4-2 coal seams is shown in Fig. 13.When the 4-1 coal seam is pushed to 120 m, the development height of the fracture zone is 9 cm above the 4-1 coal seam, and the corresponding field is 11.25 m. Afterwards, with the continuous advancement of the 4-1 coal seam, the height of the fracture zone continues to increase. When the coal seam is pushed out, the fracture zone develops to 38 m above the 4-1 coal seam, including the 3-1 coal seam and its surrounding rock strata. In the process of 4-1 coal mining, the influence on the position of the 3-1 coal area of the protected layer is increased from the beginning of the expansion deformation to the reduction of the expansion deformation and finally to the stability of the expansion deformation. The expansion deformation increases because the lower double protection layer group is mined, the ground stress at the protected layer is reduced, and a certain expansion deformation occurs in the 3-1 coal area; the gradual decrease of the expansion area is due to the continuous decline of the overlying strata under the action of gravity during the mining process of 4-1 coal, and the previous cracks are gradually compacted; the stability of expansion deformation is due to the end of the collapse of overlying strata in the stope. When the collapse shape of overlying strata reaches stability, the expansion deformation of 3-1 coal reaches a stable value.

**Fig. 14:**
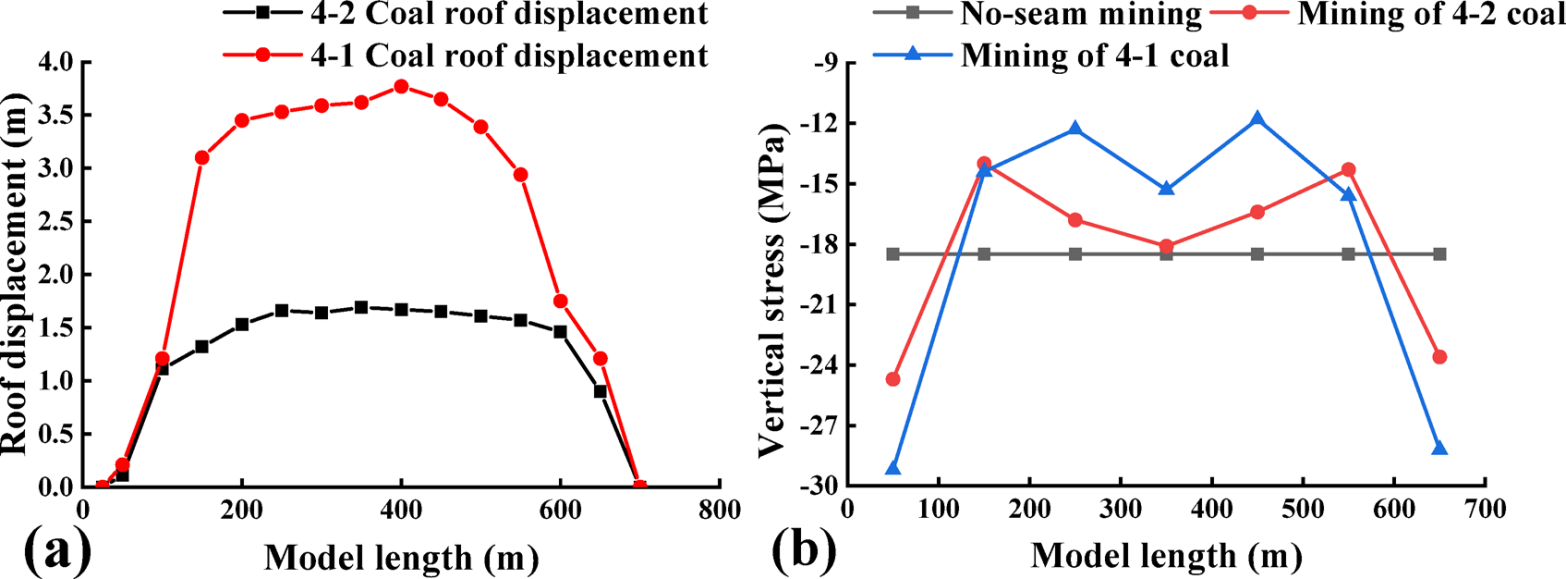
**a** 4-2 coal, 4-1 coal roof sinking volume; **b** Stress change curve of 3-1 coal.

After the model excavation was completed, the total station was used to measure the top plate of 4-1 coal seam, the overall subsidence of the top plate was large, and the working face was basically compacted, and the data read by the total station was processed to draw the map of the subsidence of the top plate of 4-2 and 4-1 coals, as shown in Fig. 14a. Read the data collected in the pressure box, and carry out certain processing on the read data, and the data processing results are shown in Fig. 14b. After the completion of 4-2 coal mining, the maximum value of stress at 3-1 coal is 24Mpa, and the maximum location occurs at the left side of 4-2 coal open cut eye and the right side of the stop line right above, at this time, the effect of pressure relief in the center of 3-1 coal is poorer; mining back to 4-1, at this time, the stress at the maximum value of stress at the left and right side of 3-1 coal rises, but the effect of pressure relief in the center of 3-1 coal is much better than only mining 4-2 coal seam.

## Conclusion

In this paper, combined with numerical simulation and similar simulation phase test, the feasibility of protective layer group in deep extra-thick coal seam mining and the stress variation law, pressure relief characteristics, expansion deformation characteristics and fracture development law of protected layer in mining process are studied. According to the results of the study, the conclusions are as follows:

1. The numerical simulation analysis shows that mining the protected layer changes the stress distribution of the original rock, thus affecting the stress distribution of the 3-1 coal. When mining the lower protected layer of 4-2 coal, the vertical stress experienced by the 3-1 coal ranges from 11.8 MPa to 18.3 MPa, initially exhibiting pressure unloading effects, with an area of pressure unloading rate exceeding 10% extending over 240 m. When mining the upper protected layer of 4-1 coal, the vertical stress experienced by the 3-1 coal is further increased, with a pressure unloading rate exceeding 10%. Subsequently, when mining the upper protective layer 4-1 coal, the vertical stress of the 3-1 coal is further reduced, with the vertical stress in the unloading area ranging between 10 MPa and 14.9 MPa, and the unloading rate exceeding 10% in an area of 340 m, resulting in a 41.6% increase in the length of the unloading area. The stress distribution curve of the 3-1 coal can be divided into four parts: the original rock stress area, the stress concentration area, the stress reduction area, and the stress restoration area.
2. The similar simulation results show that in the process of 4-2 coal mining, with the advancement of 4-2 coal, the bed separation cracks gradually appear in the rock strata above 4-2 coal, and then the cracks gradually close in the process of advancing, and the goaf of 4-2 coal is gradually compacted. At this time, the 4-1 coal seam above 4-2 coal seam has obvious bending deformation. The development height of the fracture zone is 26 m. After the mining of the upper protective layer 4-2 coal, the height of the caving zone is 7 m, and the fracture zone has been developed to the 3-1 coal area. It is about 38 m; in this process, the expansion deformation of the protected layer is divided into three processes: the expansion deformation increases, the expansion deformation decreases, and the expansion deformation is stable.
3. Numerical model calculations and similar simulation experiments have shown that the pressure unloading rate and range of the protected seam are significantly greater than those of a single protected seam. Therefore, when dealing with complex coal seams, upward mining of the protected seam can help reduce resource waste, lower the risk of coal and gas outbursts, and improve coal mining safety.

## Data Availability

The datasets generated and analysed during the current study are not publicly available due to privacy or ethical restrictions but are available from the corresponding author on reasonable request.
